# Development of a community-informed communication toolkit to prevent spread of viral illness in schools, including SARS-COV-2

**DOI:** 10.3389/fpubh.2023.1285453

**Published:** 2023-10-25

**Authors:** August Summers, Gabriela V. Calderon, Lauren M. Klein, June Wang, Janny Dinh, Tina Suliman, Erin R. Hager, Lorece Edwards, Megan E. Collins, Sara B. Johnson

**Affiliations:** ^1^Center for Communication Programs, Johns Hopkins Bloomberg School of Public Health, Baltimore, MD, United States; ^2^Department of Pediatrics, Johns Hopkins School of Medicine, Baltimore, MD, United States; ^3^Krieger School of Arts and Sciences, Johns Hopkins University, Baltimore, MD, United States; ^4^Department of Population, Family and Reproductive Health, Johns Hopkins Bloomberg School of Public Health, Baltimore, MD, United States; ^5^School of Community Health & Policy, Morgan State University, Baltimore, MD, United States; ^6^Wilmer Eye Institute, Johns Hopkins School of Medicine, Baltimore, MD, United States

**Keywords:** schools, health communication, risk communication, COVID-19, public health communication, parent engagement

## Abstract

**Introduction:**

Schools were uniquely impacted during the COVID-19 (SARS-COV-2) pandemic. We sought to elucidate how parents/guardians of elementary and middle school students in Maryland navigated the return to in-person school following remote instruction. We also sought to understand how they perceived communication about school-based COVID-19 mitigation strategies and their preferences for the content and format of public health communication about COVID-19 mitigation in schools.

**Methods:**

We engaged a community advisory board comprised of key partners and implemented a survey and focus groups.

**Results:**

Results indicated that parents/guardians wanted clearer communication about COVID-19 mitigation policies in schools and were experiencing fatigue and confusion. These insights informed the development of a tailorable communication toolkit. The toolkit was designed to (1) inform parents/guardians about the importance and effectiveness of mitigation strategies for preventing viral spread to keep children in school, (2) promote a sense of community and support, and (3) help school communication teams effectively communicate information about mitigation strategies being implemented.

**Discussion:**

We describe a process for leveraging schools as a trusted messenger, engaging school communities in the development of communication messages, and utilizing a tailorable communication toolkit in the context of shifting public health guidance and local needs. The toolkit development and dissemination process offers a model for targeting public health messaging to parents/guardians in school settings.

## 1. Introduction

School districts throughout the United States and across the world were forced to rapidly respond to the unprecedented challenges of the COVID-19 (SARS-COV-2) pandemic, which disrupted normal operating procedures. In response to the public health emergency, many schools shifted from in-person to remote learning beginning in 2020. While remote learning settings were prioritized for infection control, in-person learning has been demonstrated as the preferred setting for students’ academic success (1) and mental health (2), as well as school staff’s well-being (3).

When schools across the country welcomed students back to school buildings, the Centers for Disease Control and Prevention provided operational guidance for schools and early care and education programs to support safe in-person learning (4). The CDC continued providing periodic guidance updates in alignment with emerging scientific evidence (4). A variety of CDC-recommended mitigation strategies such as ventilation, masking, hand hygiene, and viral testing, were implemented to help reduce school-based transmission of COVID-19 among students and school staff. With this shift, there was an increased need to support parents/guardians navigating the return to in-person school and the accompanying multiple, complex, and dynamic viral mitigation policies and guidelines. While schools and school districts varied with respect to the mitigation strategies they implemented, all schools and school leaders took on the responsibility of communicating these strategies to the school community.

Risk communication is the exchange of accurate information about health risks and their severity, typically in the context of a crisis or emergency (5). Effective risk communication promotes the understanding of risk and how health-protective behaviors can reduce risk. It also facilitates informed decision-making for individuals, families, and communities; risk communication is, therefore, a significant part of public health communication. Due to the changing nature of COVID-19, changing guidance from the CDC, and the proliferation of COVID-19-related misinformation (6), effective risk communication proved challenging across a variety of settings. Building or maintaining trust and credibility as a public health messenger has been described as the “first and foremost” step in emergency effective risk communication (7).

Given the important behavioral implications of trust, effective risk communication during the COVID-19 pandemic, particularly from trusted messengers, was paramount. The clarity and timeliness of messages, openness to participatory dialogue, as well as transparency about uncertainties can all influence levels of trust; other contextual factors, such as the history of the relationship between the messenger and audience, also play a key role (7). Communication from established trusted messengers can help increase acceptance and practice of protective behaviors, especially when members of the intended audience are involved in the design and implementation of response efforts (8).

While trust is an important factor in the acceptance of protective policies and behaviors, other factors, such as perceived personal risk and susceptibility, also play a role. Behavior change theories, including the Health Belief Model (HBM), have been used to understand or predict health behaviors related to infectious disease mitigation as well as inform behavior change interventions to reduce the risk of infectious disease transmission (9–11), with demonstrated success in school settings (12–15). The HBM proposes that people’s readiness to take action is influenced by their beliefs about disease risk and their perceptions of the benefits of taking action to avoid the disease or health concern. The core constructs of the model are perceived susceptibility and perceived severity, perceived benefits and perceived barriers, cues to action, and self-efficacy. The HBM has also been successfully applied to message development promoting COVID-19 mitigation strategies (16).

The Parents And Communities as Experts (PACE) study was designed to elucidate how parents/guardians of elementary and middle school students in Maryland navigated the return to in-person school during the 2021–22 school year following statewide restrictions on in-person school during the 2020–21 school year, including their perceptions of school-based COVID-19 mitigation strategies. The study was part of a larger National Institutes of Health (NIH) Rapid Acceleration of Diagnostics-Underserved Populations (RADx-UP) Return to School initiative. The focus on kindergarten through eighth grade was identified by the RADx-UP program as an area of particular need. In the PACE study, we sought to identify cues to action and to understand what motivates parents/guardians to perceive benefits and barriers to COVID-19 preventive behavior as such.

We also sought to understand parents’/guardians’ perceptions of communication about school-based COVID-19 mitigation strategies and what public health communication regarding COVID-19 prevention strategies in schools should include. Specifically, we used the HBM to first understand parents/guardians’ acceptance of in-school COVID-19 mitigation strategies; this understanding then informed the development of a communication toolkit in collaboration with partners to support efforts to limit COVID-19 transmission in schools. In this paper, we describe the results of this formative data collection and its systematic application to the development of a communication toolkit.

## 2. Materials and methods

Consistent with the broader goals of the NIH RADx-UP initiative, of which this study was a part, we aimed to understand and address barriers to the return to in-person school, including among historically excluded groups. Consequently, the PACE study focused on 8 school districts in Maryland, chosen because they have the highest rates of students in poverty, the largest proportion of students from historically excluded racial and ethnic groups, or a location in a rural county.

**Figure 1:**
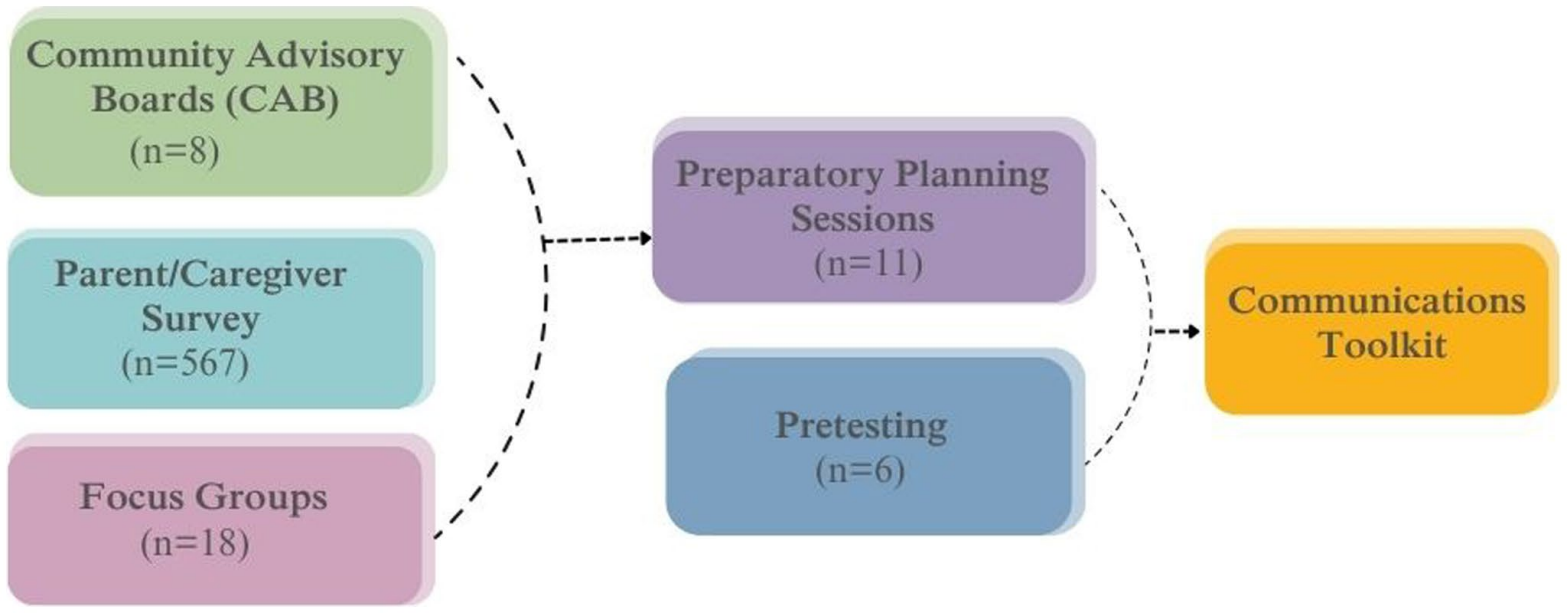
Components of the PACE study.

As illustrated in Figure 1 and detailed in Section 2.1, the PACE study involved several components. All components of the PACE study were supported by the expertise of Community Advisory Boards (CABs). CABs were comprised of community partners including parents/guardians, teachers and school staff, and school health services personnel. CAB members provided insight into study design, helped to interpret results, and shared their thoughts on COVID-19 mitigation in schools based on their daily experiences. Eight CAB meetings were held in English and Spanish from September 2021 to November 2022.

Informed by the expertise of the CAB, we fielded a web- and mail-based survey that probed parents’/guardians’ perceptions of trusted messengers for COVID-19-related information, perceptions of and attitudes toward school-based COVID-19 mitigation strategies, and barriers and facilitators to returning to and remaining in in-person school. Surveys were followed by focus groups with parents/guardians to further probe their perceptions of COVID-19 mitigation strategies in schools. We then moved to the communication toolkit design phase including a participatory toolkit planning workshop and pretesting sessions with parents and school staff to ensure that messaging included was based on needs identified by the beneficiaries of the information. Finally, informed by this formative data collection process including both qualitative and quantitative data, we developed a tailorable communication toolkit schools could use to communicate more clearly and effectively about COVID-19 mitigation in schools.

This research was approved by the Johns Hopkins School of Medicine Institutional Review Board (IRB), and participants provided informed consent.

### 2.1. Formative data collection

#### 2.1.1. Survey

The survey was available in English and Spanish and was mailed to a random sample of households likely to have children in K-8 grades in the eight target school districts between January 2022 and July 2022. In addition, a web-based version of the survey was publicized on social media, via schools, community organizations, and community events. We received a total of 567 responses. Relevant survey items included questions specific to trusted sources and acceptability of masking as a prevention strategy (used in focus group recruitment). As part of the survey, parents/guardians were asked, “*How much do you trust each of these sources to provide correct information about COVID-19*?” Respondents rated their trust on a 4-point Likert scale from “not at all” (0) to “a great deal” (3) for each of the following messengers: parent’s health care provider, child’s health provider, faith leader, close friends and family, colleagues, news, social media contacts, US government, and the child’s school officials and administrators. We previously showed that the most trusted messengers were doctors, followed by family and schools (17). Given the high levels of trust schools enjoyed and the lack of materials available to schools to support communication with families about COVID-19, we focused on developing a school-based communication toolkit.

#### 2.1.2. Focus groups

We conducted focus groups with parents/guardians to delve deeper into survey responses, probing public health mitigation, barriers and facilitators in the return to school, and potential health communication dissemination strategies. To ensure as wide a range of perspectives as possible, parents and guardians were purposively sampled for focus groups based on their responses to questions in the parent/guardian mail- and web-based survey (see above). Specifically, individuals were stratified by their response to a question on support for requiring face masks in schools to reduce COVID-19 risk. Focus groups were held virtually to promote access and participation. Between May and July 2022, 18 focus groups were conducted with 40 parents/guardians from all eight Maryland school districts. The median number of individuals in focus groups was four (range 1–8). In one case, a single participant was interviewed following the focus group guide because of scheduling challenges. Data from focus groups with parents/guardians were used to inform the specific communication objectives for the toolkit, toolkit components, tone of messages, and recommended communication channels, including key messengers.

### 2.2. Communication toolkit

We used the information gathered from the PACE Study CABs, survey, and focus groups to develop a communication toolkit that schools and school districts could tailor to their needs. The toolkit was designed to support parents’/guardians’ understanding of, trust in, and support for school-based COVID-19 mitigation strategies that schools chose to deploy.

#### 2.2.1. Participatory toolkit planning

To inform the components of the communication toolkit, parents/guardians, teachers, school staff, and other community members were invited to a participatory planning session in August 2022. The session objectives were to:

- Review key insights from surveys and focus groups
- Discuss potential communication concepts based on findings
- Prioritize the most important messages to include in communication
- Brainstorm visuals to use in communication that capture people’s attention
- Confirm the best channels to ensure the communication materials reached the school community.

Trained facilitators guided the discussion about effective communication, and participants (*n* = 11) shared their ideas based on the session objectives above. The session was 90 min in duration, and participants received a $25 gift card for their participation.

Following the participatory planning session, first drafts of communication materials were developed based on feedback and recommendations provided during the session and were guided by the HBM understanding that a person’s likelihood of practicing or supporting COVID-19 mitigation strategies is based on their perceived benefits minus the perceived barriers to taking those actions. For material development, we followed the seven C’s of effective communication: command attention, clarify the message, communicate a benefit, be consistent, create trust, cater to the head and the heart, and provide a call to action (18).

#### 2.2.2. Toolkit pretesting

School community members, including parents/guardians, teachers and staff who had participated in the planning session, were invited to pretest the drafts of communication materials and related images in an interactive process. During the pretesting sessions, we shared draft materials and alternate messaging options with participants, which were mocked up as posters to facilitate review. Pretesting sessions were administered one-on-one, held virtually, and lasted approximately 30–45 min. Participants received a $25 gift card for their participation in the pretesting activity.

In the pretesting sessions, several questions were asked to determine what changes should be made to messages and visuals to improve the materials. Some pretesting questions included: What is your gut reaction to this poster? Who do you think this poster is for? What is the main message of this poster? What emotions do you feel when you read this poster? Is there anything that you do not like about this poster? If so, what do not you like? What might a person do after seeing this poster?

We also confirmed the best communication channels to reach the school community. While drafts were displayed as mocked-up posters, the final toolkit would include communication channels suggested by participants as most effective, such as posters, flyers, social media graphics, or other formats. Following the completion of each pretesting session, notes were compiled about what worked, what did not work, and areas for improvement and clarity for communication materials based on repeated themes.

## 3. Results

### 3.1. Perceptions of communication about COVID-19 in schools

In focus group discussions, parents/guardians provided their insights on challenges and opportunities for communication about COVID-19 in schools, as well as recommendations for future school-based communication (Table 1).

**Table 1:** Sample quotes from parents/guardians participating in focus groups.

Theme: General communication Participant quotes: “Our schools started off pretty strong, you know; at the beginning of the year, they sent out lists of all the things, you know, that was gonna be implemented. And you could call and talk, and then they set up the phone system like I said, where they would call to inform you of outbreaks and whatnot. And then as the school year went on, they just kind of like – like it got to where it was an inconvenience for them. And then it was like all communication stopped soon as they dropped, like, the masks in school.” “Yeah, the lack of standardization across the board. You know so, even from school to school, let alone from county to county, there’s no consistency. And so if there’s consistency and uniform messaging as well as carrying it out so that everybody is on the same page.” “As a parent, if I’m getting mixed communications from the school district, my level of trust with regards to what I received from the school – and I know in the community that I live, this is a major issue. The trust with the school district is so broken because information goes from the Superintendent. And then when it’s dispersed in schools, you know, different schools implement it differently. And it’s like, well, who am I listening to?”
Theme: Communication channels and sources Participant quotes: “People seemed to take it better if you look like me, or if it’s coming from someone that, ‘You’re from here, you know where they are coming from. It tended to be a whole lot better because everyone could not go out into every neighborhood trying to get people information or to tell them about COVID, so you had to be concerned about cultures and all that stuff.’” “People do not trust the government information, sometimes they give us fake information just for the autocracy it benefits. So, I think parents will follow their family doctors, and town hall doctors, and all they can know for information because the government will not give you what’s going on, and so I’d say people follow their primary doctor’s information mostly.” “I just say that anybody that’s politically polarizing would be off limits because people are so staunchly on either side that if you pick someone they are like, ‘Well, I’m not going to listen to that person because they are on the opposite side.’ I would much prefer to hear and I think people would be more receptive overall to hearing from someone who wasn’t playing politics *per se*. Someone that was politically neutral I think would be the safest thing to get the most people on board.” “The most powerful medium right now is social media, the internet.” “If it is a public health campaign, I personally do not wanna see that being advertised by someone who works in an office that does not even see kids. That’s my opinion. If you get these phone calls from the superintendent, they are not in the building with the kids every single day and seeing how this affects the children. But parents are seeing how their kids are being affected. Teachers are seeing how the kids are being affected. So, I think at minimum someone within the building that has contact with the students every day would be a much more effective way to create buy-in from the families, versus a superintendent, or a communication-whatever, director or whatever those titles are. Whatever those positions are – ones that do not have direct contact with the students. It just seems very impersonal and generic, I guess.”

Many focus group participants expressed satisfaction in getting regular updates from schools. Some pointed out a lack of empathy in communication, such as the lack of acknowledgment of challenges associated with the mitigation strategies. Many participants felt that inconsistency was a recurring theme regarding mitigation strategies- there was not one singular coherent message, but rather messages from various sources which seemed to contradict each other.

Focus group participants felt that policies were not standardized across the state or even across school districts, and the guidelines for in-school mitigation strategies differed from the guidelines for non-school places. Mitigation strategies could even differ between schools in the same county. Participants stated that the way that COVID-19 recommendations were translated into school policies did not always make sense, and rules did not always seem logical. These inconsistencies led to confusion, made compliance more difficult, and, for some, led to skepticism. Participants voiced frustration with frequent changes to the rules and recommendations, especially early in the pandemic.

### 3.2. Preferred communication channels and sources

In general, focus group participants felt that as many communication channels as possible should be used to reach parents/guardians since their preferences and access to information varied. They also suggested that messaging coming from schools should be more consistent to avoid confusion from conflicting information. Some participants recommended town halls or similar formats due to opportunities for bidirectional communication, increased clarity, and a subsequently greater understanding of COVID-19-related rules and policies. In general, messages were perceived to be more trustworthy when they came from someone local, someone who was more likely to understand them and more familiar with their situation. Conversely, messages from the government were seen as less trustworthy.

### 3.3. Participatory planning session and pretesting

Parents/guardians and school staff (*n* = 102) representing all eight school districts were invited to a participatory planning session. Thirteen people responded to the invitation, and 11 people from five target school districts attended (4 parents, 7 school staff). Two participating school staff members also had a child in grade K-8. Potential communication concepts were developed based on focus group findings and were presented during the participatory session in text-only format (Table 2).

**Table 2:** Potential concepts presented during the participatory planning session.

COVID-19 fatigue is real. Here’s how you can continue to be a COVID-19 cautious parent as your child returns to school. Meaningful moments are ahead. Reduce your family’s risk of COVID-19 getting in the way. Should my kid go to school today? When, how, and why to use at-home COVID-19 rapid antigen tests and what to do if a household member or close contact has COVID-19. Understand the risks and costs of COVID-19 on students, families, school staff, including the role of vaccines in preventing severe disease, and other COVID-19 risk mitigation strategies. COVID-19 guidelines may look different in other settings. Here’s how it looks and why it’s important in schools. Let us keep our kids safe and healthy at school: address violence and bullying, prevent COVID-19 exposure, improve mental and emotional health.

Feedback on the potential communication concepts during the planning session and discussions about communication preferences reflected the insights gathered during focus groups. Participants described overall fatigue with communication about COVID-19, overwhelm with the changes in rules, and the need to express empathy toward students, parents/guardians, and staff.

“Some folks shut down at the mere mention of rules and regulations at this point.”

“…with so many changes and rules, we forgot about the human side of things; keep it light while getting messages across.”

For development and design of materials, planning session participants suggested paying attention to the literacy of the audience, providing thought-provoking and attention-grabbing images, and adding credibility to the messaging by making it clear that the key messages are coming from parents/guardians and teachers with insights into what is happening locally.

“I do think an impactful image is key, even before the main written message.”

For engagement and dissemination, planning session participants recommended involving students as ambassadors and working with community partners to disseminate information:

“Or have students themselves design the cover image - maybe an elementary and secondary version…”

“Don’t overlook existing resources and community partners who people are already comfortable with.”

When reacting to specific draft concepts (Table 2), several participants indicated their preference for communication that addressed fatigue, appealed to emotions, and was concise. Generally, there was less interest in communication that conveyed information they had heard several times already or that was perceived as lecturing. The message that resonated with most participants was one that referred to taking COVID-19 precautions to avoid missing family moments because it “appeals to the softer side” or “pulls at the heart strings.” Some participants, on the other hand, felt that the message “sugar coated” or “glazed over the point.” Participants also suggested keeping a message that COVID-19 guidelines in school settings were different than other settings as a means of transparency and justification. Specific information about COVID-19 testing was also well-received.

Participants recommended that messages about COVID-19 not be combined with other important safety topics such as school violence to prevent minimizing the importance of either message and to avoid confusion.

Six of the 11 participants who attended the participatory planning session also completed pretesting sessions, representing four school districts. Pretesting participants reviewed themes and messages that were refined based on parent/guardian and staff feedback during the participatory planning session. Themes and messages were included in draft poster format during pretesting (Table 3). Approaches included an emphasis on parent/guardian-school collaboration, addressing benefits and barriers to mitigation strategies, and presenting cues to action such as through parent testimonials and messages from school leadership. Most messaging also incorporated empathy. In one draft poster, a specific example was included to show how the latest CDC guidance on mask-wearing, isolation, and testing might be presented along with the themes and messages.

**Table 3:** Themes and messages presented on draft posters during pretesting sessions.

**Parent/guardian-school collaboration** We can thrive together with in-person learning. Let us thrive together with safe, in-person learning. Let us work together to keep kids in school. Your support and commitment to student health and safety matters, and you are not alone! COVID-19 is here to stay. Let us work together to keep kids in school. Safe, in-person learning. We’re here because of you. Did you know? In a recent school survey on COVID-19 safety, 8 out of 10 Maryland parents support wearing masks in schools. **Benefits and barriers** COVID-19 changes constantly. Our commitment is consistent. COVID-19 mitigation strategies in schools are an effective way to help limit the social, emotional, and economic impact of kids missing in-person school. Meaningful moments are ahead. Schools play a unique role in protecting our future and the community’s health. Get vaccinated and boosted. It’s the best protection against COVID-19. **Cues to action** COVID-19 has been challenging for us all. Thank you for making student safety a priority even when it’s not easy. Please continue to support the COVID-19 guidelines in [school/system name] to help keep students safe, healthy, and in-school. - John Doe, Superintendent (School leadership as trusted messenger) When Alex has COVID-19 symptoms, he gets tested. Sometimes it’s frustrating, confusing, and inconvenient, but it’s important to us. (Parent testimonial) COVID-19 has been frustrating, confusing, and inconvenient but it’s important to us to do our part to keep kids safe and in school. (Parent testimonial) The COVID-19 transmission level in [county] has changed to [level]. Safe, in-person learning is our priority and your support matters. Find additional COVID-19 guidance for our school district at [district-specific communication sources]. Stay informed and follow the COVID-19 safety guidelines in your school. Stay up to date on the COVID-19 guidelines in our schools.

During pretesting sessions, participants described preferences for photos, such as (1) a picture of real people instead of infographic-style icons, and (2) photos that reflected the demographics and/or diversity of the school community. Participants also reacted positively to photos that showed families. Families were described as a primary motivator for following COVID-19 mitigation recommendations.

Participants described a strong preference for including the phrase “in-person learning” in messages, recognizing this as a key priority for many members of school communities. Participants gave additional suggestions for specific text, headers, and taglines to keep or remove. The common preference was for an emotionally appealing message that emphasized not missing out on significant moments because of COVID-19 illness, a message that highlighted the collaboration between schools and parents/guardians in keeping students safe and healthy, as well as a photo that indicated positivity.

We categorized and summarized participant feedback from focus groups, planning sessions, and pretesting sessions for improved COVID-19 materials and communication based on the seven Cs of effective communication (18) (Table 4).

**Table 4:** Seven Cs of effective communication (18) and parent/guardian and school staff insights.

Seven Cs of effective communication	Parent and school staff insights
**Command attention** Use appealing visuals, keywords, and design elements to help attract and hold your school community’s attention.	Keywords like “in-person learning” and “safety” were suggested to get parents’/guardians’ attention. Some parents/guardians and school staff noted that they stopped reading emails that used the same templates week after week. Parents/guardians and school staff recommended thought-provoking photos.
**Clarify the message** Include a key message that is clear, direct, and concise with words and images that are easy to understand.	Mixed messages and confusion were emphasized as a key barrier. Transparency and clarity were suggested as ways to increase understanding/awareness and improve adherence.
**Communicate a benefit** Emphasize how your school community will benefit from what’s being done in your school and/or what’s being asked of them.	Providing a clear justification for why and how policies and mitigation strategies are being implemented was recommended. Benefits that were emphasized included in-person learning for students’ mental health, academics, and socialization.
**Be consistent** Provide consistent information within a material and across different communication channels and to different audiences (parents/guardians, students, staff). Use the same words across materials and communication channels to avoid confusion.	Use of multiple communication channels was recommended since information is sometimes missed and different individuals rely on different sources for information. Lack of consistency across information sources was emphasized as a major barrier. Parents/guardians and school staff suggested that people are getting information about COVID-19 recommendations from so many different places, so consistency is important.
**Create trust** Clearly present the source of the material, use trusted messengers, and be transparent and consistent. Building and maintaining trust and credibility will help encourage the school community to read and heed the information.	Trusted messengers from within and outside the school district included health providers (e.g., physicians), principals, superintendents, Chief Executive Officers, and public health professionals. Personal messages from trusted sources resonated with community experts during message pretesting.
**Cater to the heart and head** Appeal to emotions in addition to providing facts and information to help improve communication. Express empathy.	Feelings of being tired, overwhelmed, and “over it” with COVID-19 were common responses as well as parents’/guardians’ concerns about students’ social and emotional health.
**Call to action** Be clear about what the school community can do and what they are being asked to do.	Lack of knowledge and awareness of the mitigation strategies being implemented in schools was demonstrated in both survey and focus group findings among some community experts.

### 3.4. Final communication and toolkit

Community insights and perspectives from the CAB, parent survey, and focus groups guided further development of communication objectives. Informed by these quantitative and qualitative data, these objectives were to (1) keep the school community informed about the importance and effectiveness of mitigation strategies for safe in-person learning, (2) promote a sense of community and support among parents/guardians and school community members in doing their part to support a safe and healthy in-person learning environment, and (3) support schools and school district communication teams in effectively communicating information on COVID-19 mitigation strategies being implemented in their schools.

A flexible communication toolkit was determined to be an effective way to accomplish these objectives (19). Potential toolkit components were informed by strengths, needs, and opportunities identified by parents/guardians. The final toolkit included practical, actionable resources, recommendations, and materials that could be tailored to local needs. The following assets and resources were included:

- Communication guide and checklists
- Ready-to-use and customizable materials for schools
- Links to public health communication resources
- Supplemental “just in time” materials for schools and other child-serving organizations to use to promote COVID-19 safety and to address community-identified needs

While the key audience for communication messages was parents/guardians, the toolkit was designed to support the individuals and teams responsible for providing the school community with up-to-date health and safety information about COVID-19 as well as other school staff and partners who want to help parents/guardians and others in the school community feel supported and informed. The guidance included the CDC’s six principles of crisis and emergency risk communication (20).

Updated recommendations and guidance at the national and local levels may influence changes in mitigation strategies that are implemented, so adaptability in messaging and visuals allowed school districts to adjust outreach as needed, while consistently incorporating effective communication strategies. The materials were intended to supplement school districts’ current and future communications with parents/guardians. The toolkit also emphasized collaboration between schools and families to support safe environments for in-person learning.

The communication guide as part of the toolkit provided practical tips for communicating effectively about COVID-19 mitigation strategies in schools and recommended dissemination channels, based on school community feedback. It also included an overview of the seven C’s of effective communication (18), examples using specific feedback from the participatory planning session and pretesting participants, and a visual diagram of how to implement the recommendations in a sample communication material.

**Figure 2:**
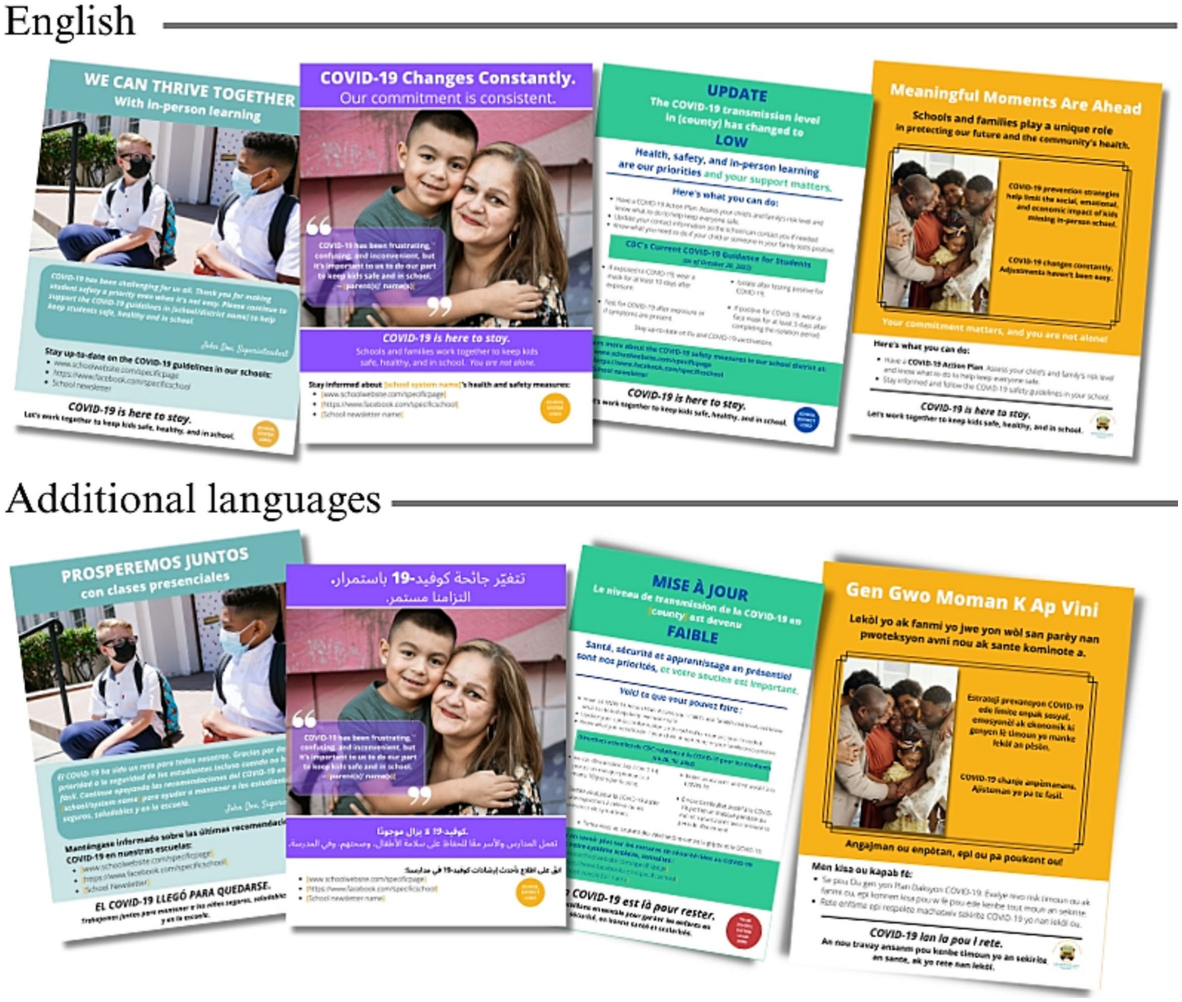
Examples of customizable and ready-to-use materials included in the communication toolkit.

The checklists were intended as tools to support the development and sharing of important information and updates to school communities. The ready-to-use and customizable materials included messages that addressed perceived susceptibility, perceived benefits, perceived barriers, and self-efficacy which were identified as most resonating and appropriate during the participatory planning session and pretesting (Figure 2). Messaging also addressed fatigue, emphasizing that individuals and communities stay vigilant. Participants recommended that multiple communication channels be used since information is sometimes missed, and different individuals rely on different sources for information. Some of the channels recommended included letters and emails sent from school sources, school newsletters, school websites, two-way dialogue opportunities (e.g., town halls), and script-based messaging (e.g., phone calls and text messages).

Because mitigation strategies varied among schools and school districts and because COVID-19 safety recommendations changed over time, the materials were designed to be easily modifiable. Thus, the toolkit included a practical guide to creating customizable materials, demonstrating how materials could be tailored to individual schools/school districts using the templates with updated messaging as needed. The graphic design platform, Canva^1^, was chosen to create customizable materials because of the its utility and accessibility to non-profit organizations. By providing modifiable templates in Canva, communication staff at individual schools and school districts would have a starting point and guidance for localizing and finalizing materials.

A list of existing public health communication resources was provided in the toolkit, which included additional communication guidelines, sample messages and scripts, and additional ready-to-use materials. Additional ready-to-use materials for schools and other child-serving organizations to use to promote COVID-19 safety and to address community-identified needs were also provided (Figure 2).

### 3.5. Communication toolkit dissemination

Parent/guardian and school staff recommendations directly informed the dissemination of the communication toolkit. As a first step, the study team developed a list of over 25 people including superintendents, school district Chief Executive Officers, school principals, administrators, and heads of communication departments across the 8 Maryland school districts using school district website information. An announcement about the upcoming availability of the communication toolkit was emailed to this list at the beginning of October 2022, followed by another email at the end of the month announcing publication with a link to the final study report and communication toolkit. School leaders were directed to access the resources at the study website where resources would be added and updated over time.^2^

Additional dissemination strategies included outreach and presentations to state and school district health councils, providing an overview and demonstration of the toolkit. We also created a video that introduced the toolkit and walked users through ways they could customize the materials.

Based on participant feedback, as materials were provided to school districts for dissemination, we encouraged school district leaders and communication teams to be transparent and consistent across platforms and sources (schools vs. school districts) to help increase trust among school community members. This might mean including clear justification if and when they chose to implement mitigation strategies and providing consistent information to parents/guardians, students, and school staff about mitigation strategies and positive COVID-19 cases, when feasible. CAB members requested that materials be available in additional languages. The final materials were translated into Spanish, French, Haitian Creole, Swahili, Arabic, and Nepali. To date, the communication toolkit has been viewed or downloaded 6,000 times and is being iteratively refined in response to user feedback.

## 4. Discussion

To date, few interventions at the school level have focused on communicating effectively with parents/guardians about public health emergencies. While many studies have focused on the efficacy of specific COVID-19 mitigation strategies in schools and their combination, the effectiveness of these strategies depends almost entirely on individuals’ willingness to adopt and maintain them (21, 22). This requires careful attention to both risk communication best practices and community engagement. We developed a communication toolkit to support parents’/guardians’ understanding of and participation in COVID-19 mitigation strategies using insights gathered from focus groups, parent surveys, Community Advisory Boards, and participatory planning and pretesting. The communication toolkit is a community-informed public health strategy to increase understanding and support for school mitigation strategies for COVID-19. Participatory planning sessions and pretesting helped to ensure that messaging and materials developed were acceptable, easy to understand, and clear on important action steps for staying informed of the schools’ mitigation strategies, knowing the importance of supporting those strategies to help prevent the spread of viral illness. Understanding the perspectives of parents/guardians and community members about the role of schools in COVID-19 mitigation led to a data-driven tool and community-driven approach to reach parents/guardians through schools. This approach can be replicated in other settings and contexts. The results demonstrate potential strategies for leveraging schools as a trusted messenger in emergency response situations, engaging the school community in the development of communication messages, and recognizing the utility of providing a tailorable communication toolkit in an evolving information environment.

This communication toolkit reflects the understanding that schools are an important messenger of public health information. Schools and school leaders are perceived by parents/guardians as trustworthy messengers of COVID-19 information (6, 17). The communication toolkit accounted for this finding by tailoring resources for the most trusted school personnel and allowing for flexibility in messaging to reach various populations.

Because updated recommendations and guidance at the national level such as from the CDC (23) and local agencies influence mitigation strategies that are ultimately implemented in schools, the flexible design of the toolkit emphasized the need to tailor the content to current recommendations and audiences. The adaptability allowed schools to reach and relate to various audiences who could then use information as a cue to public health-related action.

To be effective, the optimal approach to risk communication is in response to timing and context. In the later stages of emergency response, for example, when the sense of urgency has declined, it is necessary to combat fatigue with adherence to recommendations, emphasizing that communities must stay vigilant and maintain protective behaviors (8). Effective risk communication is especially imperative and challenging in the context of a novel virus.

Several international and national health organizations have provided materials, tools, or guidance for communicating about COVID-19 (24–28), including in the school setting (29–31). We built upon these and included recommendations for how to tailor messages locally and make updates as COVID-19 guidelines change and provided customizable templates in a user-friendly and accessible graphic design platform.

Recommended messaging in the customizable and ready-to-use materials in the toolkit was also grounded in the HBM. For example, messages addressed the perceived barriers to communication identified during focus groups, such as fatigue or feeling overwhelmed with the quantity of COVID-19 information and guidelines, lost trust and credibility, lack of transparency and consistency, and confusion as a result of receiving mixed messages from the schools or school districts. Cues to action provide a stimulus for decision-making. External cues to action suggested were recommendations and reminders from trusted messengers, such as school administrators, including alerts or updates about the current community transmission levels that impact school-level decisions to update or reiterate mitigation strategies.

This approach, integrating formative research and community participation in communication toolkit development, helped incorporate insights and feedback from multiple perspectives, including both parents/guardians and school staff who had varying levels of involvement in the school’s existing communication with the school community. This approach was well-suited for the objectives of the PACE study, particularly because COVID-19 recommendations and implementation in schools looked different than in other settings. Hearing directly from the audience members about what was and was not working in communication efforts in school-specific settings was imperative. The approach was also ideal because of the opportunity to build trust and show empathy to parents and school staff who had been managing ongoing challenges throughout the pandemic.

While developed in response to COVID-19, this toolkit can be adapted for use in response to viral spread in general. If replicated, this approach could represent a model strategy for developing and supporting health communication outreach in school settings. This process is not limited to emergency public health response but is relevant to non-crisis contexts and a wide range of issues related to health behavior change. It can be replicated to address other public health priorities. This model can also be applied to other settings where understanding context or setting-specific factors, trust-building, and empathy are important.

### 4.1. Limitations

A key strength of the PACE Study was the engagement of parents/guardians and staff in the development of the communication toolkit; however, those who participated in study activities and feedback sessions may have had a more positive attitude about public health communication in general, in comparison to those who did not choose to participate.

The study was developed in a single state, Maryland, in which remote learning was mandated for public schools during the Spring of 2020 and, later, mitigation was relatively robust (32). The perspectives of study partners and participants likely reflect their experiences in remote learning and school-based prevention. Other states and districts may have provided different feedback about priorities. Nonetheless, the approach used here could be adapted for diverse geographical settings.

Community Advisory Board engagement, focus groups, a participatory planning session, and pretesting sessions took place over 12 months (September 2021 to September 2022). There were many changes to COVID-19 mitigation guidelines, case rates, and other factors in that interval; therefore, some community insights became outdated and less relevant over time. Moreover, although multiple individuals and stakeholders participated in this process, attendance varied across opportunities to engage.

While some insights were likely applicable across all school districts, it may have been beneficial to develop communication toolkits at the individual school district level if time and resources were available. While the communication toolkit provided both ready-to-use materials and recommendations for tailoring the customizable materials to local needs, school districts may have benefited from having additional materials that were both ready-to-use and localized to their school district.

An ideal next step in the PACE study would be to assess the utilization of the toolkit and the impact on self-protective behaviors. Data on the impact of risk communication on self-protective behaviors are lacking (33). While data are being tracked on the number of visits to the PACE study website, utilization of the toolkit and any subsequent impact on behavior change or attitudes is unknown. It would also be beneficial to determine any changes in self-efficacy to communicate with parents/guardians in a public health emergency among school district staff who were responsible for outreach, based on their use of the toolkit. School staff in these roles were not involved in pretesting.

### 4.2. Conclusion

Schools are an important and trusted messenger of public health information. Communication methods that are adaptable and tailorable are needed for schools to reach and relate to various audiences who may then use information as a cue to public health-related action, especially in the context of shifting guidance and risk communication efforts that demand a high degree of trust. To inform the development of the communication toolkit, we undertook substantial community-partnered formative work. Based on feedback from school community members, we aimed to provide resources and materials to support clear and concise communication from those identified as trusted messengers within the school district. The toolkit development and dissemination process represents a model for developing public health messaging for parents/guardians in school-based settings. Data- and community-driven approaches can be effective in leveraging the existing relationship between parents/guardians and schools, where these groups collaborate to meet student health and safety needs.

## Data availability statement

The original contributions presented in the study are included in the article/supplementary material, further inquiries can be directed to the corresponding author.

## Ethics statement

The studies involving humans were approved by Johns Hopkins School of Medicine Institutional Review Board. The studies were conducted in accordance with the local legislation and institutional requirements. The participants provided their written or oral informed consent to participate in this study.

## Author contributions

AS: Conceptualization, Writing – original draft, Visualization. GC: Project administration, Writing – review & editing, Investigation, Supervision. LK: Investigation, Writing – review & editing. JW: Investigation, Writing – review & editing. JD: Data curation, Investigation, Writing – review & editing. TS: Writing – review & editing. EH: Conceptualization, Funding acquisition, Supervision, Writing – review & editing. LE: Conceptualization, Funding acquisition, Writing – review & editing. MC: Writing – review & editing. SJ: Conceptualization, Funding acquisition, Investigation, Supervision, Writing – review & editing.

## Footnotes

^1^ www.canva.com

^2^ https://schoolhealth.jhu.edu/covid19_resources/pace-study/
